# Capecitabine-Induced Genital Hand-Foot Syndrome Treated With Topical Tacrolimus

**DOI:** 10.7759/cureus.57570

**Published:** 2024-04-03

**Authors:** Bryan L Chan, Tina Wang

**Affiliations:** 1 Internal Medicine, Huntington Hospital, Los Angeles, USA; 2 Oncology, Huntington Hospital, Los Angeles, USA

**Keywords:** capecitabine-induced, topical tacrolimus, palmar-plantar erythrodysesthesia, hand-foot syndrome, balanitis

## Abstract

We describe a rare case of capecitabine-induced palmar-plantar erythrodysesthesia (PPE), or hand-foot syndrome (HFS), involving the genitals, which resolved with tacrolimus therapy, in a patient with cT3dN3 stage IIIc moderately differentiated proximal rectal adenocarcinoma who was undergoing neoadjuvant chemotherapy. Given its severe impact on the quality of life, HFS often requires independent local anti-inflammatory treatment and subsequent dose delay and/or modification of the patient’s chemotherapy. We believe that our findings in this report can aid clinicians in the early recognition and management of capecitabine-associated HFS resulting in balanitis, as prompt treatment may reduce morbidity and avoid prolonged interruption of chemotherapy in these patients.

## Introduction

Palmar-plantar erythrodysesthesia (PPE), also known colloquially as hand-foot syndrome (HFS), is a cutaneous toxic reaction associated with many common cytotoxic agents such as capecitabine, cytarabine, and docetaxel. The mechanism of action of HFS is not fully understood, but it is believed to occur on the palms and soles due to the transportation of drugs from the eccrine sweat glands to the stratum corneum, where the drugs accumulate and ultimately cause a local cutaneous reaction [1]. This reaction is often characterized initially by paresthesia, which is followed by erythema, edema, and blistering of the hands and feet with resultant epidermal necrosis [2,3].

HFS classically presents in the hand and feet, and its presentation in the genital region (causing balanitis) is extremely rare and not well characterized. Given the debilitating nature of the disease and the significant effects on the quality of life, early recognition and treatment of the condition is essential to avoid long-term interruption of potentially outcome-modifying chemotherapy [3-5]. In this report, we discuss a unique case of capecitabine-induced HFS involving the genitals, which resolved with local tacrolimus therapy after initial treatment failure with topical steroids and discontinuation of capecitabine.

## Case presentation

**Figure 1:**
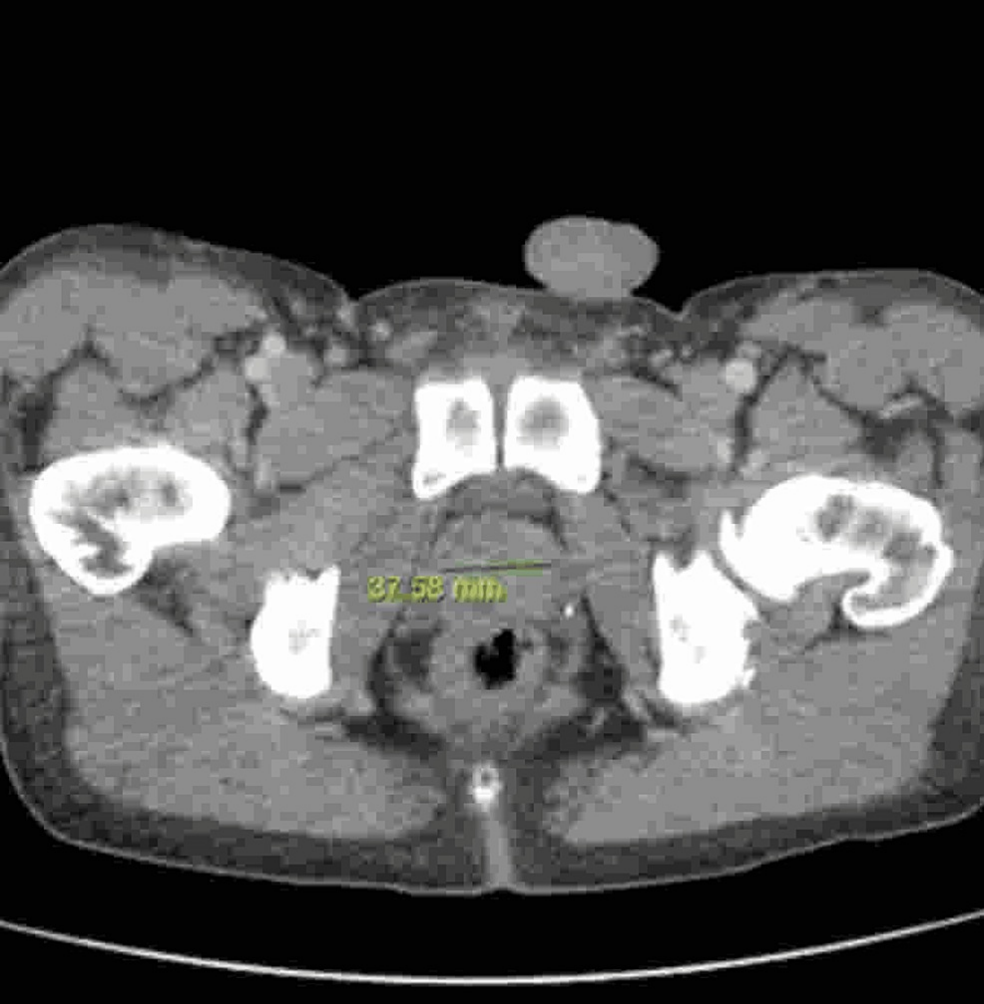
CT abdomen and pelvis (axial view) with contrast The image shows mild-moderate focal concentric thickening of the rectal wall, a hypodense lesion in the left hepatic lobe (1.7 cm), and a posterior perirectal mesenteric nodule (1.4 cm) CT: computed tomography

The patient was a 38-year-old male with a past medical history of attention-deficit disorder who presented with six months of worsening gastrointestinal symptoms including increased urgency, frequency, loose stools, and tenesmus. He denied any abdominal or rectal pain but reported two episodes of hematochezia during his six months of symptoms. He denied any nausea, vomiting, fevers, or chills. The patient underwent a colonoscopy, which showed an infiltrative and ulcerative 7 cm mass in the proximal rectum causing a partial obstruction. Biopsy of the mass on the same day demonstrated moderately differentiated rectal adenocarcinoma in the setting of adenoma with high-grade dysplasia. Staging CT abdomen and pelvis with contrast (as opposed to PET-CT) revealed a 1.7 cm hypodense lesion in the left hepatic lobe, mild-moderate focal concentric thickening of the rectal wall, and a posterior perirectal 1.4 cm mesenteric nodule (Figure 1). There was no evidence of metastatic disease in the pelvis. The case was subsequently discussed at the admitting hospital's multidisciplinary tumor board, which led to a consensus to pursue neoadjuvant chemotherapy first, followed by chemotherapy with radiation, and then a surgical resection. Per patient preference, the decision was made to start CAPEOX as opposed to FOLFOX given the former's greater general tolerability.

**Figure 2:**
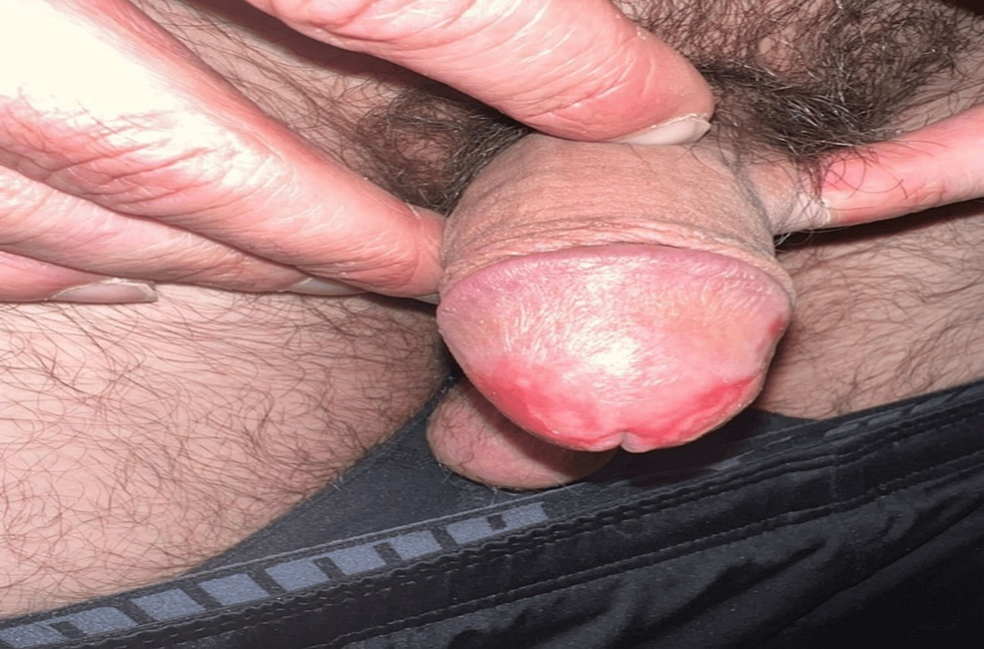
Capecitabine-induced palmar-plantar erythrodysesthesia of the patient’s genitalia, predominantly involving the glans penis

The patient began treatment with CAPEOX and initially experienced side effects of mild fatigue, abdominal bloating, and diarrhea. He denied any skin changes or sensitivities in his hands or feet. He did, however, notice increased penile irritation and erythema without visible ulceration about a week after the first cycle. After cycle 2, he began to notice dysuria in addition to penile erythema and tenderness. He was treated with Macrobid for empirical management of UTI (after an unremarkable urinalysis and culture) and referred to Urology. Urology prescribed topical antifungal cream and topical steroids, but his symptoms continued to worsen. A general sexually transmitted disease panel was ordered, which returned negative for HIV, G/C, and HSV. After the second cycle, the patient was also noted to have mild grade 1 erythema and pain in the hands and feet. Cycle 3 of CAPEOX was dose-reduced, but he continued to have worsening penile symptoms with significant ulcerations in the glans penis, severe pain, and erythema with any movement (Figure 2). He also developed peeling in bilateral hands. 

**Figure 3:**
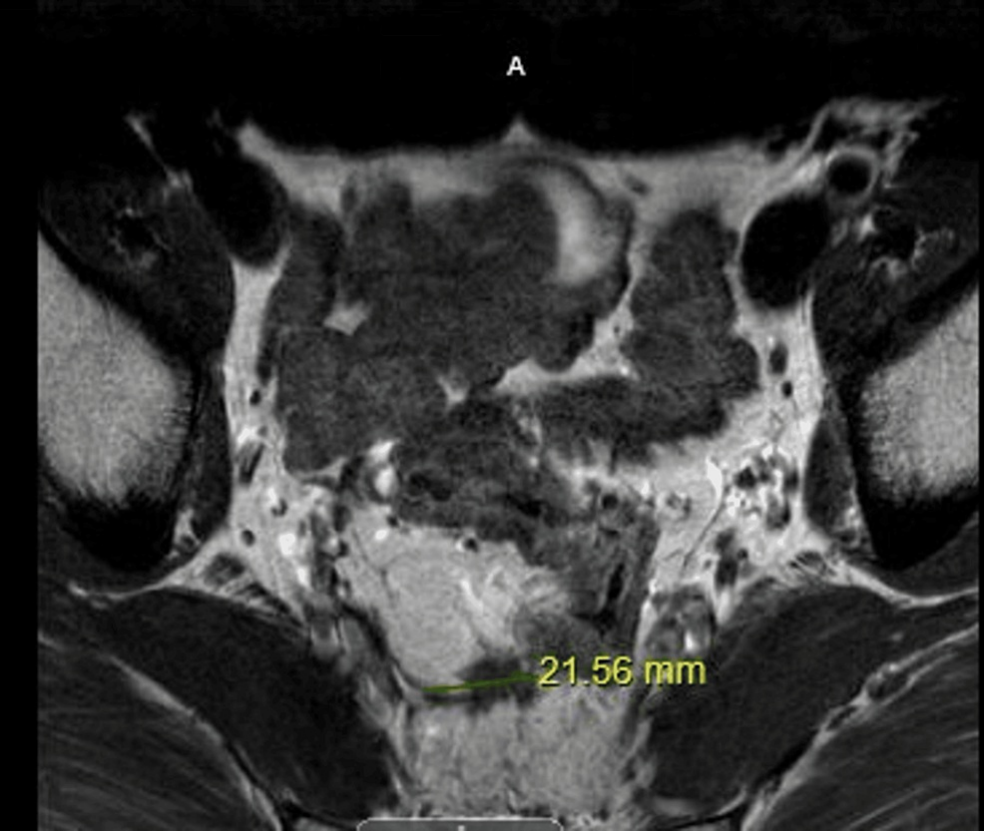
MRI with IV contrast, T2 axial view The image shows 4.3 cm upper to mid-rectal carcinoma with adjacent diffuse posterior perirectal tumor infiltration and posterolateral mesorectal lymphadenopathy MRI: magnetic resonance imaging

Cycle 4 of chemotherapy was delayed for several weeks with an improvement of symptoms in the palms of the hands, but a progression of balanitis. Although a change in the chemotherapy regimen was considered, the patient opted for a dose reduction of the current chemotherapy after receiving counseling from the primary oncologist. He was eventually evaluated by dermatology, who prescribed topical tacrolimus (given the steroid-sparing, anti-inflammatory property of the drug) along with mupirocin, which led to prompt improvement in the patient's balanitis. He was able to resume cycles 4, 5, and 6 of dose-reduced CAPEOX with the resolution of genital symptoms. Biopsies of the lesion were deferred given stark improvement after chemotherapy discontinuation and the application of the topical agent. He subsequently completed concurrent chemoradiation with full-dose capecitabine therapy without recurrence of penile symptoms, and with mild HFS symptoms in the extremities. A recent outpatient follow-up MRI of the rectal mass showed an appropriate clinical response to CAPEOX (Figure 3).

## Discussion

Given the insidious onset of symptoms that manifested initially in the genitals, the differential diagnosis of the patient’s clinical presentation was broad and included capecitabine-induced HFS, allergic reaction (including allergic contact dermatitis), autoimmune disease, fixed-drug eruption, contact dermatitis, and sexually-transmitted infections such as syphilis, HSV, and G/C. The other etiologies were excluded based on the lack of response to topical steroids, antifungals, antibiotics, and negative STD/infectious workup. Given the temporal relationship of the lesions with the chemotherapy, as well as the stark improvement with dose discontinuation, we can likely attribute the patient's symptoms to capecitabine-induced balanitis, which coincided with the subsequent development of HFS in his hands and feet.

Capecitabine acts as an antimetabolite, which is broken down to fluorouracil, also known as 5-FU, which ultimately interferes with the production of DNA by blocking the action of thymidylate synthase. This process thereby disrupts cancer cells from dividing, and capecitabine-based neoadjuvant chemoradiation is the standard treatment for locally advanced rectal carcinoma [6]. However, the associated toxicity may often affect gastrointestinal, hematologic, and integumentary systems; HFS is a common adverse reaction, often involving focal irritation, erythema, and peeling of the hands or feet [2,7]. Prior studies state that as many as three in four patients develop some degree of HFS, although HFS involving the genitals is notably rare and has only been reported in a handful of prior case studies; it is even rarer in patients who have not yet received radiotherapy [3,8]. According to the National Cancer Institute (NCI) grading of HFS toxicity, higher grades (grade 3) represent more significant adverse cutaneous effects and symptomatology; our patient developed grade 2 toxicity in the feet and hands [9]. Although the NCI grading system does not classically describe HFS in the genital region, the patient’s ulcerative features and severe pain interfering with ambulation and daily living would likely qualify as a grade 3 toxicity at the least, with many considering as possibly a grade 4 toxicity [8].

In addition, perhaps the most unusual feature of this case is the patient’s positive response to topical tacrolimus after treatment failure with topical steroids. Tacrolimus was chosen by the dermatologist due to the anti-inflammatory properties of the drug without the adverse side effects of steroids, which may be particularly harmful when applied to sensitive areas such as the face or genitals. Typically, the first-line treatment for HFS involves dose reduction or interruption of the offending agent in addition to local supportive care, with symptoms often resolving after two weeks to a month of discontinuation. More recently, topical Voltaren has been proposed as a cost-effective way to reduce the risk of HFS for patients treated with capecitabine, by as much as 75% [3,9,10]. Topical steroids, local cooling, and moisturization therapy have all been shown to have some efficacy in the treatment of HFS, with topical sildenafil also proposed to be effective in relieving symptoms of HFS in some individuals [10]. Topical tacrolimus, due to its anti-inflammatory properties, has been utilized as a treatment for multiple inflammatory cutaneous lesions such as atopical dermatitis, lichen planus, psoriasis, and lupus erythematosus. It is associated with a lack of traditional adverse effects linked to corticosteroid treatment, such as skin atrophy and systemic absorption [5,9,10]. Our case report is the first of its kind to describe the resolution of HFS symptoms on the genitals with primary tacrolimus topical therapy in addition to temporary chemotherapy discontinuation and preventative dose reduction.

## Conclusions

We presented a unique case of capecitabine-induced balanitis in a patient on neoadjuvant chemotherapy alone, without concurrent radiotherapy, with prompt resolution after primary treatment with topical tacrolimus and chemotherapy discontinuation. Besides recognizing HFS outside of its typical location in the extremities, our report also demonstrates the novelty and effectiveness of topical tacrolimus as a treatment option in clinical situations where steroids, dose-reduction, and even treatment hiatus have faltered. Future studies should aim to more methodically assess the application of this drug beyond this extremely rare presentation.
